# A Blessing in Disguise

**Published:** 1897-07

**Authors:** 


### EDITORIAL DEPARTMENT.

F. E. DANIEL, M. D., Editor.
S. E. HUDSON, M. D., Managing Editor.
A. J. SMITH. M. D., Galveston, Associate Editor.

EDITORIAL STAFF:

PROF. J. E. THOMPSON, M. D., Texas Medical College, Galveston; Surgery.
PROF. WM. KEILLER, M. D., Texas Medical College, Galveston; Obstetrics and Gynecology.
PROF. DAVID CERNA, M. D., Texas Medical College, Galveston; Therapeutics.
PROF. A. J. SMITH, M. D., Texas Medical College, Galveston; Medicine.
DR. R. H. L. BIBB, City of Mexico; Foreign Correspondent.
Published Monthly at Austin, Texas, by Drs. Daniel and Hudson. Subscription price $2.00 a year in advance.

Eastern Agents: The Monihan-Fairchild Special Agency, 24 Park Place, New York City.

Official organ of the West Texas Medical Association, the Houston District Medica Association, the Austin District Medical Society, Brazos Valley Medical Association the Galveston County Medical Society, and several others.

### A BLESSING IN DISGUISE.

Well, the agony is over. The Texas legislature finally adjourned without passing any practice act, thus again disappointing quite a number of physicians of the State. The *Texas Medical News* grows very angry over it, and abuses the Speaker of the House in unmeasured terms, and unjustly blames him, we think, for the failure. The *News* calls him "traitor" and all sort of hard names, and alleges that the speaker "promised Dr. Field that he would recognize him to call up the bill," and didn't do it.

Well, its no fight of ours, the *News* and Mr. Speaker for it. It is right funny tho' how our genial contemporary swells with wrath and threatens Mr. Speaker with the "influence" of the doctors! The "influence" does not seem, so far, to have been an available quantity,—at least in securing medical legislation. Dr. McLaughlin may be able, however, to crystalize it into a bolt of wrath with which to smite Mr. Dashiel. We'll see later.

The writer cannot help thinking that after all, it is best for the profession that the act did not pass. I believe had it done so the time would have come when all would have regretted the inconsiderate step,—recognizing homeopathy. It was inconsiderate, and hastily done; and done too by a mere tythe of the medical profession. There were less than one hundred members present at the meeting when the bill was adopted, and some thirty of them were new men who had just joined at that meeting. It cannot, therefore, be claimed that the adoption of the bill recognizing homeopathy as it did, was the sentiment of the profession of Texas. Nine-tenths of the physicians of the State were absent, and knew nothing of the action, except what they read in the TEXAS MEDICAL JOURNAL. There is no way of ascertaining their sentiments; but we venture the statement that if a personal canvas had been made or could be made, and the subject fairly presented, the proposition to thus take the homeopaths into our loving embrace as equals, recognizing them as a school of medicine—thus tacitly confessing that the medical profession is only another school or pathy,—it would have been overwhelmingly defeated. The profession as represented by the State Medical Association realize so forcibly the need of a proper act regulating the practice that in their zeal to procure it they go too far. They sacrifice too much for a questionable expediency. We would have lived to regret it had the bill passed.

The pretext that we must recognize "the other schools" in order to have the bill come within the constitutional limits is the sheerest *stuff*. The present law distinctly stipulates that a diploma, to be lawful, must emanate from a medical college recognized by the American Medical Association. That certainly does not recognize any "school," and yet, as we have before stated, it has been pronounced constitutional; has stood the test.

Hope springs eternal in the medical breast. Defeated this time we will succeed next. In the interval between now and next legislature if we will go to work actively to organize the medical profession locally,—in every village and neighborhood where there are six doctors or more, and let each organization send up its delegates to a full meeting, and then let us formulate a bill which will make no distinction in practitioners; but require all to conform to one standard; in the meantime educate the people on the subject; make them see the matter as we do, and especially make the representatives understand the question before they leave home; then, and not till then may we hope to secure a suitable medical act.

The friends of the Association bill—the homeopathic triumph—relied implicitly on the presence in the legislature of several doctors; they felt sure that these doctors would have influence enough to secure the passage of the bill. Here they reckoned without their host; they had forgotten to ask them if they consented to this affiliation? They did not consent to it, and hence in some quarters where the committee expected support, they found opposition. Dr. S. J. Morriss, of Cass county, in the Senate, and Dr. A. C. Oliver, in the House, were active in their opposition (and, of course came in for their share of the venom of the *Texas Medical News*). These are both old experienced physicians, physicians of the old school of ethics, trained and taught all their lives to put a value upon the name of physician, and to guard the honor and dignity of their calling. It was a mistake to suppose that such men—whose opinion had not been asked—would support such a mongrel bill. They could not just see the wisdom of sacrificing honor, dignity and pride in the profession, to a mistaken idea of expediency. When men of their character and standing refuse their support to a medical bill, recognizing, as they do, the necessity for a medical law, there must be some grave defect in it. There are no two men in the profession who would have been more rejoiced to see a good medical law enacted than these gentlemen; but they could not swallow "an act," as Dr. Morriss styled it in a letter to the *Texas Medical News*, "to degrade and disgrace the medical profession." That the nine physicians in the legislature could have secured the passage of the bill had they been united, there is little doubt. Had it been the right kind of bill they would have been united, and their influence would have been felt. That they—some of them—opposed the bill is an indication, or proof rather, of the correctness of our position as above stated, that the rank and file of the profession are opposed to "homologating" with this or any other form of quacks. Hence, we suggest that in the next legislature let there be a large representation of doctors, and let us put before them a bill that they can endorse, and we will get it through.

